# Studies on the extended spectrum beta lactamases activity in isolates from diabetes patients

**DOI:** 10.1186/1471-2334-12-S1-P86

**Published:** 2012-05-04

**Authors:** AL Dorothy Kalyani, T Palaniappan, V Mohan, T Sundararaj

**Affiliations:** 1 Dr. Mohan’s Diabetes Specialities Centre, Gopalapuram, Chennai, India; 2 Meenakshi Ammal Dental College, Maduravoyal, Chennai, India; 3 Jasmn Laboratory, Jasmn Education & Research Foundation, Perungudi, Chennai, India

## Background

Extended spectrum beta-lactamases (ESBLs) are enzymes produced by some gram negative bacilli that mediate resistance to extended-spectrum cephalosporins and aztreonam. ESBLs are commonly recognized in a variety of *Enterobacteriacea*e and *Pseudomonas aeruginosa* isolates. This study focused on the prevalence of ESBL producing strains from diabetes patients and their antimicrobial correlation.

## Methods

ESBL activity studied in different gram negative bacteria isolated from xxx urine samples were subjected to antimicrobial susceptibility testing by Kirby-Bauer method as per CLSI guidelines antimicrobials agents (Cefpodoxime, Ceftazidime, Cefotaxime, & Ceftriaxone) selected for testing along with combinations of antimicrobials Cefoperazone sulbactam (CFS), Piperacillin-tazobactam (PT) Amoxyclav (AC) were compared for their ability to detect ESBL producers phenotypically.

## Results

Among 4446 samples processed 3426 showed ESBL activity in different gram negative bacilli 85% in *E.coli*, 7% *Klebsiella pneumoniae*, 0.5% *Klebsiella oxytoca*, 2.3% *Pseudomonas aeruginosa*, 0 .5% *Citrobacter koseri*, 1% in *Proteus mirabilis*.

## Conclusion

There is a significant increase in the prevalence of ESBL *E.coli* when compared to other gram negative isolates. Detection of ESBL by phenotypic method in the absence of molecular testing is considered as timely, affordable and appropriate measure in deciding the antibiotic therapy.

